# Development of a functional composite for the evaluation of spinal and bulbar muscular atrophy

**DOI:** 10.1038/s41598-022-22322-w

**Published:** 2022-10-19

**Authors:** Tomonori Inagaki, Atsushi Hashizume, Yasuhiro Hijikata, Shinichiro Yamada, Daisuke Ito, Yoshiyuki Kishimoto, Ryota Torii, Hiroyuki Sato, Akihiro Hirakawa, Masahisa Katsuno

**Affiliations:** 1 Department of Neurology, Nagoya University Graduate School of Medicine, 65 Tsurumai-Cho, Showa-Ku, Nagoya, 466-8550 Japan; 2 Department of Clinical Research Education, Nagoya University Graduate School of Medicine, 65 Tsurumai-Cho, Showa-Ku, Nagoya, 466-8550 Japan; 3 Department of Clinical Biostatistics, Graduate School of Medical and Dental Sciences, Tokyo Medical and Dental University, 1-5-45 Yushima, Bunkyo-ku, Tokyo, 113-8519 Japan

**Keywords:** Neurology, Neurodegeneration

## Abstract

This study aimed to develop a functional measurement that combines quantitative motor evaluation index of various body regions in patients with spinal and bulbar muscular atrophy (SBMA). We assessed subjects with SBMA and healthy controls with quantitative muscle strength measurements and functional scales. We selected tongue pressure, grip power, % peak expiratory flow (%PEF), timed walking test, and % forced vital capacity (%FVC) as components. By combining these values with Z-score, we created a functional composite (SBMA functional composite: SBMAFC). We also calculated the standardized response mean to compare the sensitivity of SBMAFC with that of existing measurements. A total of 97 genetically confirmed patients with SBMA and 36 age- and sex-matched healthy controls were enrolled. In the longitudinal analysis, the standardized response mean of SBMAFC was larger than that of existing rating scales. Receiver operating characteristic (ROC) analysis demonstrated that the SBMAFC is capable of distinguishing between subjects with early-stage SBMA and healthy controls. SBMAFC is more sensitive to disease progression than existing functional rating scales and is a potential outcome measure in clinical trials of SBMA.

## Introduction

Spinal and bulbar muscular atrophy (SBMA) is an adult-onset, X-linked hereditary neuromuscular disease caused by polyglutamine repeat expansion in the *androgen receptor* gene^1,2^. The principal clinical symptoms of SBMA are progressive muscle weakness and atrophy resulting from lower motor neuron degeneration accompanied by muscular involvement^3–5^. The progression of neurological deficits in SBMA is usually very slow, with an average interval of more than 20 years between the onset of symptoms and death^6^.

Although there are no treatments for SBMA that have shown efficacy in confirmatory clinical trials, the development of disease-modifying drugs has become an active area in clinical research, facilitated by the rapid growth of studies elucidating the molecular pathogenesis^7^. Since an expected efficacy of such therapies is to slow the disease course, there is pressing need for reliable, easy-to-use scales that can detect subtle clinical changes over short periods. Several functional scales including the Spinal and Bulbar Muscular Atrophy Functional Rating Scale (SBMAFRS) have been utilized as outcome measures in clinical trials of SBMA, however, the sensitivity and reliability of these scales are limited^8,9^. On the other hand, objective muscle strength measurements including grip power and the 6 min walking test have the advantage of highly objectivity and quantitativity. However, they also have the disadvantage of solely representing the physical function of a certain body region (e.g., grip power for upper limb function). An idea to overcome these issues is the development of a composite that integrates objective measurements for partial motor function and, thereby, represent total physical function.

In the present study, we aimed to develop a disease-specific functional composite to evaluate the physical function of the patients with SBMA.

## Methods

### Standard protocol approvals, registration, and participant consent

This study was conducted in compliance with the Helsinki Declaration, the Ethical Guidelines for Human Genome/Gene Analysis Research, and the Ethical Guidelines for Medical and Health Research Involving Human Subjects by the Japanese government and was approved by the Ethics Review Committee of Nagoya University Graduate School of Medicine. All participants provided written informed consent prior to study participation.

### Patients and healthy controls

Patients with SBMA were recruited if they met the following inclusion criteria: a clinical diagnosis of SBMA with more than one motor symptom (muscle weakness, muscle atrophy, bulbar palsy, and hand tremor); confirmation of *androgen receptor* CAG repeat expansion (> 38 repeats); age from 20 to 80 years at the time of informed consent; no experience with medications with disease modifying effects, such as leuprorelin acetate; and the ability to attend ambulatory hospital visits. Heathy controls (HCs) were recruited if they met the following inclusion criteria: age from 20 to 80 years, able to attend ambulatory hospital visits, and no neurological diseases. We also included age-matched male HCs through recruitment of volunteers. Subject characteristics, including age, disease duration, and CAG repeat expansion, were collected as baseline characteristics. Disease duration was defined as the age when subjects first noticed their subjective muscular weakness of any body regions. All subjects were Japanese and were observed at Nagoya University Hospital between March 2013 and January 2021.

### Functional scales

SBMAFRS is a disease-specific validated functional rating scale that measures total physical function in patients with SBMA^8^. SBMAFRS includes five domains: bulbar-related (5 items: speech, control of salivation, swallowing, tongue, puffing cheeks); upper limb-related (2 items: writing, eating action); trunk-related (4 items: dressing activity, arising from a sitting position, arising from a supine position, bowing); lower limb-related (2 items: walking, stairs); and respiration-related (1 item: breathing).

The Amyotrophic Lateral Sclerosis Functional Rating Scale (ALSFRS) is a validated questionnaire-based scale that measures total physical function in patients with ALS performing ADL^10^. A revised version of this scale, ALSFRS-R, was generated to improve the disproportion of weighting to the limbs and bulbar system, as compared to respiratory dysfunction. The ALSFRS-R has been translated into Japanese and validated^11^. ALSFRS-R, as well as SBMAFRS assesses the function of bulbar, upper limb, trunk, lower limb, and respiratory musculatures.

### Quantitative functional measures

Based on the results of previous clinical studies on SBMA, we selected the tests as objective measurements to be integrated in the functional composite: tongue pressure for bulbar function; grip power for upper limb function; timed walking test for lower limb function; peak expiratory flow for truncal function; and vital capacity for respiratory function.

Tongue pressure measures the maximal tongue force during the oral stage of swallowing. Decrease of tongue pressure has been detected in SBMA patients even at early stages without subjective dysphagia^12^. A high reproducibility and a strong association of tongue pressure assessment with bulbar function have also been shown. In the preset study, we used a digital tongue pressure measurement device (JMS Co., Ltd., Hiroshima, Japan), and asked the participants to compress the balloon of a disposable intraoral pressure probe upward onto their palates for 7 s using the maximum voluntary effort of the tongue. We recorded tongue pressures 3 times at 1 min intervals and adopted the maximum pressure recorded as the maximal tongue pressure (kPa) for each time. We adopted the average of the maximal tongue pressures for our analyses, unless otherwise stated^12–14^.

Grip power has been utilized to evaluate upper limb function in varying clinical studies of SBMA, as well as those of other neuromuscular disorders^15–19^. In the present study, we measured grip power using an electronic hand dynamometer as previously described.^9^ The examinees were instructed to keep their elbows at 90 degrees, their forearms in neutral rotation, and their wrists not flexed or pronated. The measurements were performed twice for each side, and the larger value was adopted as one’s grip power in each side. The average value was adopted as one’s grip power.

There are several methods to evaluate ambulation performance of subjects with SBMA: 6-min walk test, 2 min walk test, and timed walking test^19–21^. Patients with SBMA often present with proximal lower limb involvement and fatigue. Therefore, the other measurements, such as the 30-sit to stand test or 6 min test, might be suitable for this composite measurement. However, we adopted the 15-feet timed walking test as an objective measure of lower limb function, as this measurement requires shorter time and distance and has less chance of fall than other tests. Additionally, timed walking test is acceptable for patients with decreased gait function and/or fatigability. In the present study, we calculated a walking speed (km/hr.) during participants walk over 15 feet, so that higher scores indicate better performance as well as the other components because a past study indicated that 6 min walking capacity correlated well with the reciprocal number of the time required for walking 15 feet^22^.

We used % forced vital capacity (%FVC) and % peak expiratory flow (%PEF) of pulmonary tests as objective measures of respiratory and truncal function, respectively^23,24^. The %FVC is a standard index of respiratory function of neuromuscular disease, and our previous studies demonstrated that %PEF is strongly correlated with truncal subscores of SBMAFRS and ALSFRS-R^25^. A pulmonary function test was performed for all subjects using a spirometer (FUDAC-77; FUKUDA DENSHI, Tokyo, Japan), which calculated and recorded FVC, forced expiratory volume in 1 s (FEV1.0), the ratio of FEV1.0 to FVC, and PEF. The predicted values of FVC and FEV1.0 were calculated using Baldwin's Equation^26^ and Berglund's Equation^27^, respectively. The PEF is defined as the maximum expiratory flow per minute, which can be used to measure how fast a subject can exhale, as well as to judge the strength of the expiratory muscles and the condition of the large airways. The %PEF is calculated from regression equations for predicting PEF in the Japanese population. The subjects sit in a chair with a backrest and are instructed to inhale as deeply as possible, and then exhale through a mouthpiece as quickly as possible, with their noses occluded.

The Approximate time to complete these quantitative measurements was about 20 min.

### Development of functional composite using Z-score (SBMAFC)

To combine various quantitative motor measurements with different units, we utilized Z-score, a standardized score that enables quantification of an individual’s physical performance in comparison with the average performance in the population. We calculated Z-score as the number of standard deviation units that a patient's score differs from the average score.

To develop a functional composite, the spinal and bulbar muscular atrophy functional composite (SBMAFC), we adopted the following five objective quantitative measures: tongue pressure, grip power, walking speed of timed walking test, %PEF, and %FVC. We transformed these values to Z-scores using the mean and standard deviation of the values of healthy controls. For example, A Z-score of bulbar function in a certain person is calculated according to the following formula. ${Z}_{bulbar}=({X}_{i bulbar}-\overline{{X }_{i bulbar}})/\sqrt{Var{(X}_{i bulbar})}$ We calculated the SBMAFC of each subject by taking the total of five Z-scores: Z_composite_ = Z_bulbar_ + Z_upper limbs_ + Z_trunk_ + Z_lower limb_ + Z_respiration_. A better score on one or all components compared to the mean results in a higher SBMAFC score, whereas a worse score compared to the mean results in a lower overall SBMAFC.

### Comparison of quantification ability for the early-stage subjects with SBMA

Quantitative evaluation of the subjects with very mild symptoms is essential to conduct clinical studies because recent developments of disease-modifying therapeutics target patient populations at preclinical to early stages of neurodegenerative diseases including SBMA. In the present study, we analyzed sensitivity and specificity of SBMAFC, ALSFRS-R, and SBMAFRS by comparing the subjects with early-stage SBMA and HCs using receiver characteristic curve (ROC) analysis. We defined the subjects with early-stage SBMA as patients whose disease duration was within five years and with the total score of ALSFRS-R of 47 or higher because an ALSFRS-R score of 47 is greater than the mean score minus two times SD in healthy controls (Table 1). The subjects for this subgroup analysis were extracted from the main cohort of the present study.

**Table 1:** Baseline characteristics.

	Subjects with SBMA (n = 97) Mean ± S.D. (range)	Healthy controls (n = 36) Mean ± S.D. (range)
Age at examination (yrs.)	51.9 ± 10.9	53.9 ± 8.0
	(25–76)	(38–68)
Disease duration (yrs.)	9.0 ± 6.5	–
	(0–29)	
CAG repeat length in AR gene	47.6 ± 3.9	–
	(42–58)	
SBMAFRS	42.1 ± 6.9	55.7 ± 0.5
	(19–56)	(54–56)
ALSFRS-R	41.1 ± 3.9	47.8 ± 0.4
	(28–48)	(46–48)
Tongue pressure (kPa)	18.0 ± 6.8	41.6 ± 7.8
	(5.3–38.3)	(26.5–61.9)
Grip power (kgw)	21.2 ± 6.7	45.0 ± 6.4
	(5.9–44.6)	(28.0–59.8)
%FVC	99.4 ± 15.0	112.0 ± 11.1
	(47.5–133.1)	(92.8–142.7)
%PEF	84.4 ± 19.4	116.4 ± 22.4
	(8.1–135.6)	(78.6–158.8)
15 feet timed walking (km/hr.)	4.86 ± 1.76	7.68 ± 1.96
	(0.552–9.356)	(4.52–13.92)
Serum creatinine (mg/dL)	0.49 ± 0.16	0.86 ± 0.14
	(0.23–0.94)	(0.49–1.21)

AR, androgen receptor; SBMAFRS, spinal and bulbar muscular atrophy functional rating scale; ALSFRS-R, the revised amyotrophic lateral sclerosis functional rating scale; %FVC, % forced vital capacity; %PEF, % peak flow; SD, standard deviation.

### Statistical analysis

We used descriptive variables such as the mean and standard deviation to summarize the quantitative measures. To show score distribution, scatter plots were utilized in the SBMA and HC groups. Pearson correlation coefficients were calculated between SBMAFC and the existing functional rating scales, SBMAFRS and ALSFRS-R. To compare the diagnostic ability of outcome measurement, SBMAFC, ALSFRS-R, and SBMAFC, the ROC analysis was utilized. The area under the ROC curves ± standard error of the mean was provided and compared. In the longitudinal analysis, a standardized response mean (SRM) was also calculated using the ratio of the mean score change to the standard deviation of score change, and used as an index of the effect size for a direct comparison among the outcome measures. Values of 0.20, 0.50, and 0.80 were considered to represent small, moderate, and large changes over time, respectively^28^. Sample size estimation was performed with the observed variability in score changes for a hypothetical intervention that could reduce progression rates by 10%–100% with power (1-β) set at 0.8 and α at 0.05. Statistical analyses were performed using SPSS Statistics 27.0 (SPSS Japan, Inc., Tokyo, Japan) except for the ROC analysis. The ROC analysis was performed using software EZR 1.41 (Saitama Medical Center, Jichi Medical University, Japan)^29^. A *p*-value of < 0.05 was considered as statistically significant.

## Results

### Baseline characteristics

**Figure 1:**
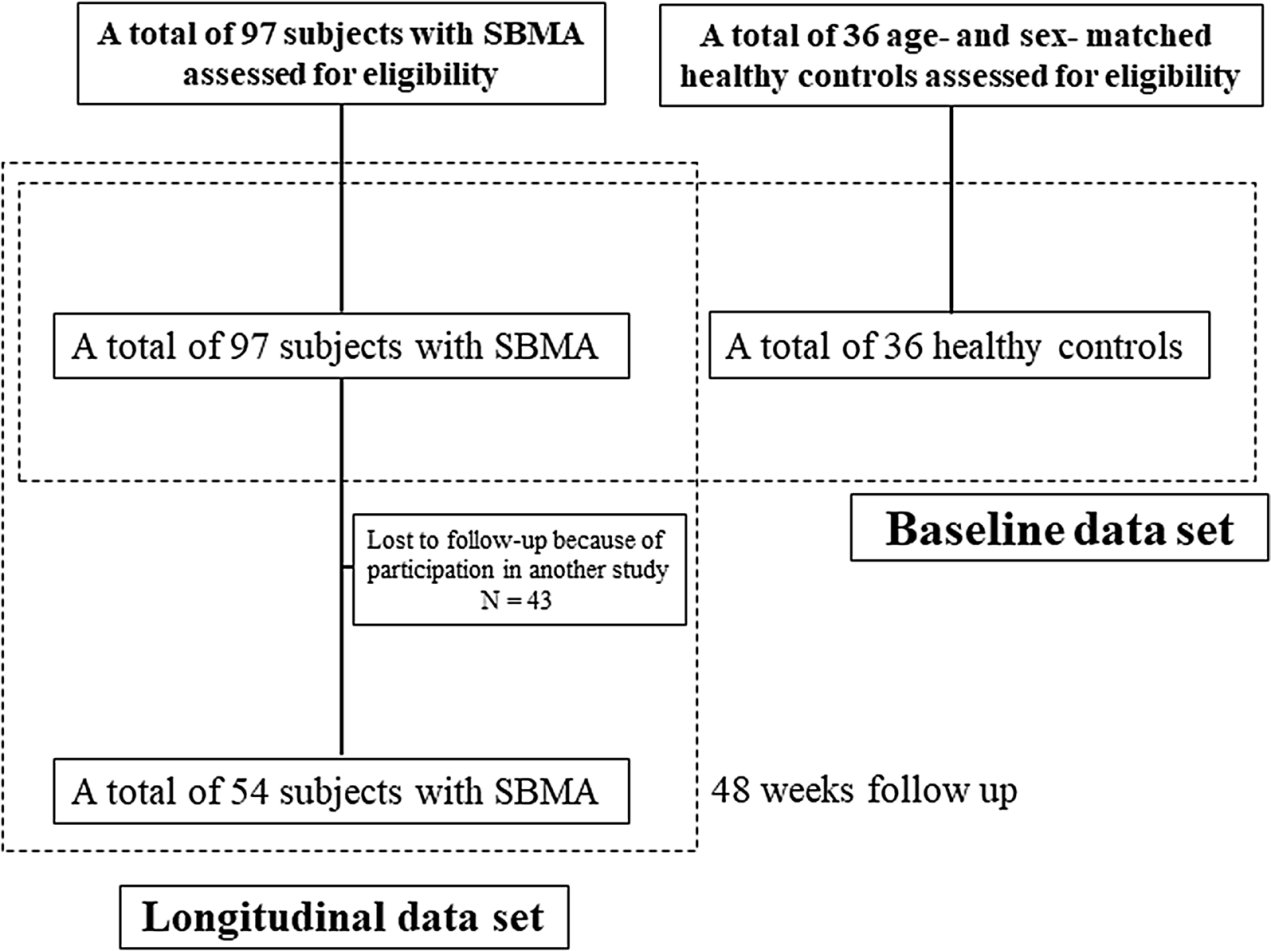
Flowchart of study population enrollment A total of 97 subjects with SBMA and 36 age- and sex-matched healthy controls with no diagnosed neurological disorders were enrolled. A total of 54 patients were followed up for 48 weeks. A total of 43 subjects at 48 weeks follow-up were lost because they participated in the other interventional clinical studies.

A flowchart of study participant enrollment is shown in Fig. 1. Briefly, we enrolled a total of 97 subjects with SBMA and 36 age- and sex-matched HCs with no diagnosed neurological disorders, from whom baseline data were obtained (Table 1). In the longitudinal study, 54 SBMA patients were followed up for 48 weeks. A total of 43 out of 97 subjects were lost to follow-up at 48 weeks because they participated in the other interventional clinical studies. However, background characteristics did not substantially differ between the groups (baseline-only group and 48-week follow up group) (Table 1, Supplementary Table 1). The characteristics of the subjects with SBMA, such as age at examination, disease duration, or CAG repeat length on the *androgen receptor* gene, were similar with those of previous studies^6,8,30,31^. Based on the means and standard deviations of healthy controls at baseline (Table 1), we computed a composite score, SBMAFC, with the following formula: SBMAFC = (Tongue Pressure (kPa)—41.6)/7.84 + (Grip Power (kgw)—45.0)/6.4 + (%PEF—116.4)/22.4 + (Timed Walking speed (km/hr)—7.7)/2.0 + (%FVC—112.0)/11.8.

### Relationship between SBMAFC and functional scales

The value of SBMAFC correlated well with those of SBMAFRS and ALSFRS-R. To investigate the internal validity of SBMAFC, relationships of each component of SBMAFC and total value of SBMAFC were analyzed (Supplementary Fig. 1). The values of SBMAFC components in subjects with SBMA correlated well with the total score of SBMAFC at baseline.

### Comparison of quantification ability of SBMAFC, ALSFRS-R, and SBMAFRS in subjects with early-stage SBMA

**Figure 2:**
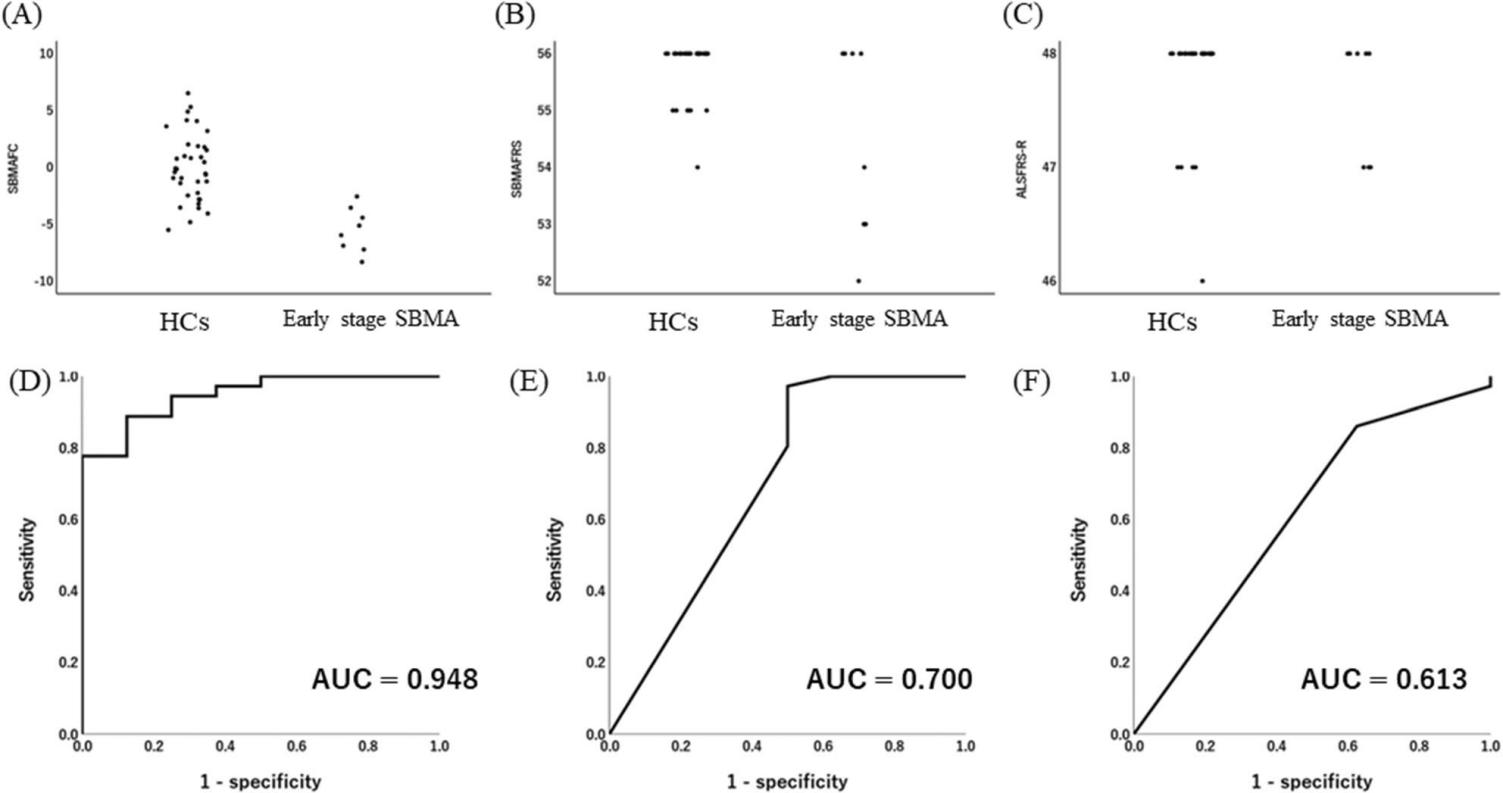
SBMAFC, SBMAFRS, and ALSFRS-R in early-stage SBMA (**A–C**). Score distribution of SBMAFC, SBMAFRS, and ALSFRS-R. A ceiling effect in healthy controls (HCs) was not shown for SBMAFC, but was clearly detected for SBMAFRS (**B**) and ALSFRS-R (**C**). (**D–F**). A receiver operator characteristic (ROC) analysis of SBMAFC (**D**), SBMAFRS (**E**), and ALSFRS-R (**F**).

In the present study, a total of 8 of 97 subjects with SBMA were defined to be at an early stage. The mean age at evaluation and disease duration were 38.6 ± 14.1 and 2.0 ± 2.2, respectively. The score distributions of the outcome measures and the ROC curves indicated that the area under the curve of SBMAFC was larger than that of ALSFRS-R or SBMAFRS (SBMAFC, 0.948; SBMAFRS, 0.700; ALSFRS-R, 0.613, respectively) (Fig. 2). These results indicate that SBMAFC is advantageous for detecting subtle symptoms in subjects with early-stage SBMA.

### Longitudinal change of SBMAFC

**Figure 3:**
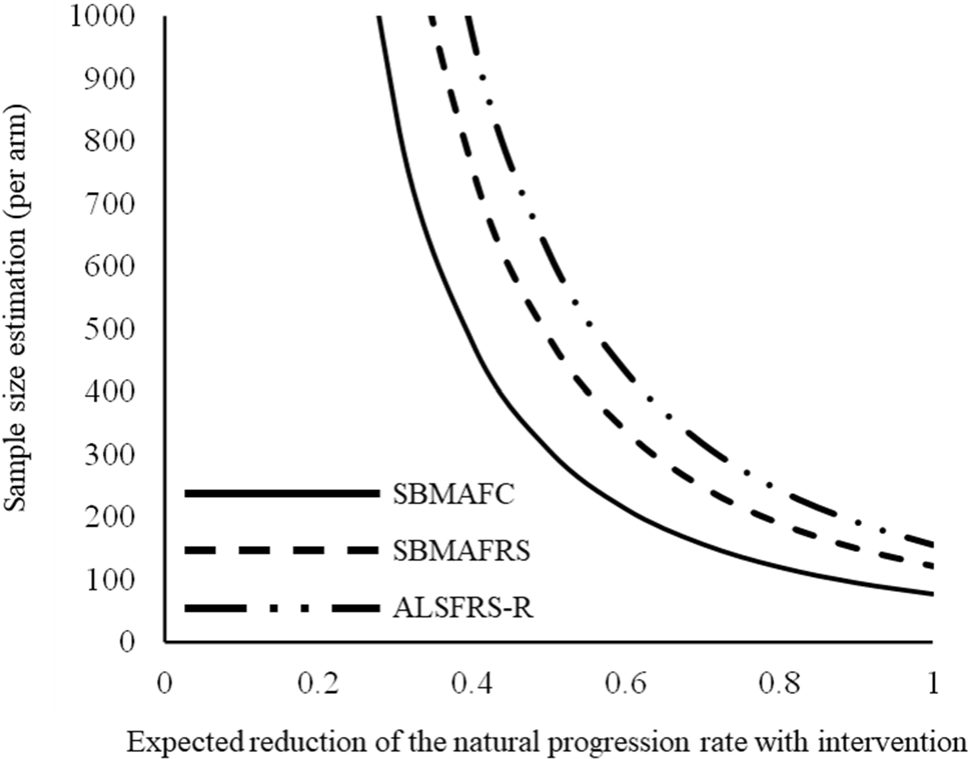
Sample size estimation Sample size estimation performed with the observed variability in score changes for a hypothetical intervention indicated that the responsiveness was better in the SBMAFC than in the existing functional scales, SBMAFRS and ALSFRS-R.

To clarify how sensitive SBMAFC is to disease progression, we analyzed the longitudinal change of SBMAFC. The results showed that the SRM of the SBMAFC was larger than that of SBMAFRS or ALSFRS-R in 48-week follow-up (Table 2). The SRM of SBMAFRS was larger than that of ALSFRS-R, in agreement with a previous study showing the higher sensitivity of SBMAFRS over ALSFRS-R regarding longitudinal changes^7^. These results indicate that responsiveness is greater in the SBMAFC than in the existing functional scales, SBMAFRS and ALSFRS-R. Sample size estimation based on this longitudinal analysis was the lowest for the SBMAFC, followed by the SBMAFRS and the ALSFRS-R, confirming that the SBMAFC is a sensitive clinical measure that detects disease progression over time (Fig. 3).

**Table 2:** Longitudinal change of outcome measurements in 48-week follow-up.

	Baseline (n = 54) Mean ± S.D	48 weeks follow-up (n = 54) Mean ± S.D	Longitudinal change Mean ± S.E	SRM *
**Items of composite measurements**				
Tongue pressure (kPa)	18.8 ± 7.2	17.9 ± 3.4	− 0.57 ± 0.48	0.1683
Grip power (kgw)	21.7 ± 7.0	21.1 ± 7.4	− 0.61 ± 0.32	0.2761
PEF%	85.2 ± 18.6	84.9 ± 19.6	− 0.37 ± 1.70	0.0308
Walking speed (km/hr.)	5.05 ± 1.89	4.54 ± 1.91	− 0.51 ± 0.15	0.4956
%FVC	100.0 ± 14.0	99.5 ± 13.8	− 0.51 ± 0.61	0.1196
SBMAFC	− 10.34 ± 3.27	− 10.83 ± 3.39	− 0.487 ± 0.154	0.4532
**Existing measurements**				
SBMAFRS	43.2 ± 6.2	42.0 ± 7.6	− 1.24 ± 0.49	0.3593
ALSFRS-R	41.6 ± 3.9	40.9 ± 4.4	− 0.74 ± 0.33	0.3176

*SRM (Standard response mean) is an index of the effect size for direct comparison among the outcome measures calculated as the ratio of the mean score change to the standard deviation of score change. A larger number indicates high sensitivity.

%VC, % vital capacity; %PEF, % peak flow; SBMAFC, spinal and bulbar muscular atrophy functional composite; SBMAFRS, spinal and bulbar muscular atrophy functional rating scale; ALSFRS-R, the revised amyotrophic lateral sclerosis functional rating scale; S.D., standard deviation; S.E., standard error of the mean.

## Discussion

In the present study, we created a functional composite of SBMA and evaluated its validity and longitudinal change. The composite, SBMAFC, is a simple, quantitative, and practical clinical measurement that combines bulbar, upper limb, truncal, lower limb, and respiratory functional measures. The values of SBMAFC correlated well with the scores of the existing rating scales, SBMAFRS and ALSFRS-R. SBMAFC discriminates subjects with early-stage SBMA from normal subjects more efficiently than the existing rating scales. Furthermore, our longitudinal analysis revealed that the SRM of SBMAFC was greater than that of SBMAFRS and ALSFRS-R, indicating a high sensitivity of the composite to disease progression.

Clinical endpoints in clinical trials should be valid, reliable, and sensitive. A simple example is the impact of treatment on a hard endpoint such as death or pneumonia^32^. Such simple, robust outcomes are desirable, but might not be achievable within a limited trial period for slowly progressive neuromuscular diseases including SBMA. Thus, motor functional measurements are utilized more commonly in the field of clinical studies for neuromuscular diseases. There are two types of outcome measurements to assess motor function that are mutually complementary. The first type is the functional rating scale, such as ALSFRS-R for amyotrophic lateral sclerosis or SARA for spinocerebellar ataxia^9,33^. These scales can evaluate total physical function, but are only semi-quantitative indexes. They are also susceptible to subjectivity of raters and examinees^34^. The other type is quantitative measure, such as grip power or 6 min walking test. These measurements are objective and less susceptible to placebo effects^34^; however, they can only assess the partial physical function of a specific body region. SBMAFC combines the advantages of both types of measures. The composite is an objective measure that reflects total physical function with a high sensitivity to disease progression. Our results suggest that SBMAFC can be used for evaluating natural history and efficacy of intervention even at very early stages of SBMA.

Functional composites have been created for various neurological disorders. For example, the multiple sclerosis functional composite (MSFC), which consists of quantitative tests of arm and hand function (9-hole peg test), cognitive function (paced auditory serial addition test, PASAT), leg function, and ambulation (Timed 25-Foot Walk)^35^. MSFC has been widely used to evaluate motor function of subjects with MS in various clinical trials of disease-modifying drugs^36,37^. Spinocerebellar Ataxia Functional Index (SCAFI) and Composite Cerebellar Functional Score (CCFS) are composites for spinocerebellar ataxias. The former combines 8 m walking time, 9-hole peg test, and PATA repetition rate, while the latter integrates 9-hole peg, click, tapping, and writing tests^38,39^. Both composites are more sensitive than SARA to detect the efficacy of tested drugs in clinical trials^40^. The response of SBMAFC to therapy should be validated in future clinical trials.

In addition to a large longitudinal change, SBMAFC has an advantage in detecting a mild motor deficit at an early stage of SBMA. Several studies clearly indicate that biochemical alterations precede the onset of subjective symptoms in most neurodegenerative diseases. This view has been most explicitly demonstrated in Alzheimer’s disease, in which amyloid deposition emerges more than 20 years prior to the onset of dementia^41^. Regarding SCA, a multicenter longitudinal study reported mild ataxia and gray matter atrophy in the brainstem and cerebellum in carriers of SCA1 and SCA2 mutations^42^. Pre-symptomatic carriers of mutations causing Huntington’s disease and frontotemporal dementia demonstrate subtle motor and cognitive deficits together with brain atrophy^43,44^. Our previous study indicates that serum levels of creatinine show a decline with disease progression of SBMA, which starts more than 10 years before the onset of subjective weakness and is followed by the emergence of hand tremor, a prodromal sign of the disease. In the present study, the values of SBMAFC, but not those of functional scales, were substantially different between subjects with early-stage SBMA and HCs. Unlike ALSFRS-R or SBMAFRS, there were no ceiling effects in SBMAFC in HCs. This likely underlies the high sensitivity of SBMAFC in early-stage SBMA. Further studies that enroll early stage or prodromal phase subjects with SBMA are essential to confirm sensitivity in earlier stage of SBMA.

The present study has several limitations. We selected five components of SBMAFC based on the results of previous reports without rigorous analysis of candidate outcome measures, since limited numbers of measurements have been examined in published clinical studies on SBMA. We may need to develop more quantitative measurements and include them into create a composite in future studies. Some markers, including serum creatinine or motor unit number estimation evaluated by electrophysiological tests, are reported to be promising biomarkers to reflect clinical severity, but we did not include such indices for creating SBMAFC. It is needed to develop other outcomes by adopting blood and/or electrophysiological biomarkers as composite measurement components. Although subsets of participants were followed for up to 48 weeks in the present study, many subjects were lost during the longitudinal study because of enrollment in the other interventional clinical studies. We cannot exclude the possibility that this selection bias affected the results of the present study. Furthermore, we did not analyze test–retest data, therefore our study cannot assure the reproducibility of SBMAFC.

In conclusion, the functional composite SBMAFC is more sensitive to the disease progression of SBMA than existing functional scales, including SBMAFRS and ALSFRS-R, and has potential to be utilized in future clinical trials. In addition, the SBMAFC may detect subtle symptoms at an early stage of disease, and, thus, is a potential outcome measurement of clinical trials of disease-modifying therapies for SBMA.

## Supplementary Information

Supplementary Information.
