# Supplementary material for: Development of a functional composite for the evaluation of spinal and bulbar muscular atrophy
Source: Sci Rep. 2022 Oct 19;12:17443. doi: 10.1038/s41598-022-22322-w (PMC9581920; doi:10.1038/s41598-022-22322-w)
Supplement: Supplementary file 1 — Supplementary Information. [file 41598_2022_22322_MOESM1_ESM.pdf]

Supplementary data

Development of a functional composite for the evaluation of spinal and bulbar muscular atrophy

Tomonori Inagaki[1#], Atsushi Hashizume[1, 2#*], Yasuhiro Hijikata[1], Shinichiro Yamada[1], Daisuke Ito[1], Yoshiyuki Kishimoto[1], Ryota Torii[1], Hiroyuki Sato[3], Akihiro Hirakawa[3], Masahisa Katsuno[1, 2*]

1. Department of Neurology, Nagoya University Graduate School of Medicine, Nagoya, 466-8550, Japan
2. Department of Clinical Research Education, Nagoya University Graduate School of Medicine, Nagoya, 466-8550, Japan
3. Department of Clinical Biostatistics, Graduate School of Medical and Dental Sciences, Tokyo Medical and Dental University, Tokyo, Japan.

[#]These authors contributed equally.

Contents:

Supplementary Table 1

Supplementary Figures 1

Supplementary Table 1. Subject background of the 48 week follow-up group

	48 week follow-up group (n = 54)
Age at examination (yrs.)	52.0 ± 11.6 (25 - 76)
Disease duration (yrs.)	9.0 ± 7.0 (0 - 29)
CAG repeat length on AR gene	47.9 ± 4.3 (42 - 58)
SBMAFRS	43.2 ± 7.1 (27 - 56)
ALSFRS-R	41.6 ± 3.9 (33 - 48)
Tongue pressure (kPa)	18.4 ± 7.2 (5.3 - 38.3)
Grip power (kgw)	21.7 ± 7.0 (5.9 - 44.6)
%FVC	100.0 ± 14.0 (62.0 - 133.1)
%PEF	85.3 ± 18.6 (34.2 - 135.6)
15 feet timed walking (km/hr)	5.05 ± 1.89 (0.55 - 8.72)
Serum creatinine (mg/dL)	0.48 ± 0.13 (0.25 - 0.74)

AR, androgen receptor; SBMAFRS, spinal and bulbar muscular atrophy functional rating scale; ALSFRS-R, the revised amyotrophic lateral sclerosis functional rating scale; %FVC, % forced vital capacity; %PEF, % peak flow

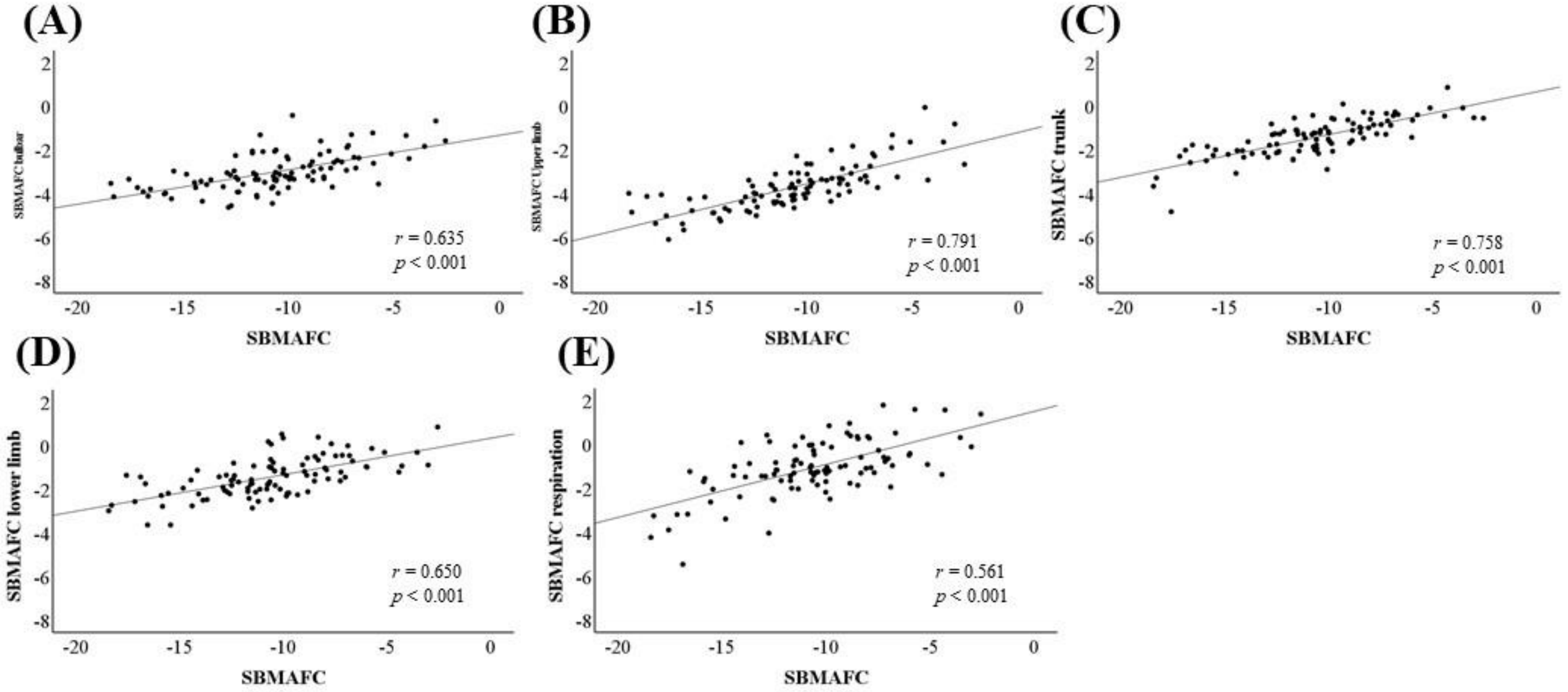

Supplementary Figure 1. Relationship between each component and total value of SBMAFC

The correlation between the Z-score of each component of SBMAFC and total value of SBMAFC (A–E). The total score of SBMAFC correlates well with each domain of SBMAF
